# Genetic evidence for efficacy of targeting IL-2, IL-6 and TYK2 signalling in the prevention of type 1 diabetes: a Mendelian randomisation study

**DOI:** 10.1007/s00125-024-06267-5

**Published:** 2024-09-13

**Authors:** Tea E. Heikkilä, Emilia K. Kaiser, Jake Lin, Dipender Gill, Jaakko J. Koskenniemi, Ville Karhunen

**Affiliations:** 1 Institute of Biomedicine, Research Centre for Integrative Physiology and Pharmacology, University of Turku, Turku, Finland; 2 Faculty of Social Sciences, Tampere University, Tampere, Finland; 3 Department of Medical Epidemiology and Biostatistics, Karolinska Institutet, Stockholm, Sweden; 4 Department of Epidemiology and Biostatistics, School of Public Health, Imperial College London, London, UK; 5 Health Informatics Institute, Morsani College of Medicine, University of South Florida, Tampa, FL USA; 6 Research Unit of Population Health, Faculty of Medicine, University of Oulu, Oulu, Finland; 7 Research Unit of Mathematical Sciences, Faculty of Science, University of Oulu, Oulu, Finland; 8 MRC Biostatistics Unit, School of Clinical Medicine, University of Cambridge, Cambridge, UK

**Keywords:** Co-localisation, IL-2, IL-6, Mendelian randomisation, TYK2, Type 1 diabetes

## Abstract

**Aims/hypothesis:**

We aimed to investigate the genetic evidence that supports the repurposing of drugs already licensed or in clinical phases of development for prevention of type 1 diabetes.

**Methods:**

We obtained genome-wide association study summary statistics for the risk of type 1 diabetes, whole-blood gene expression and serum protein levels and investigated genetic polymorphisms near seven potential drug target genes. We used co-localisation to examine whether the same genetic variants that are associated with type 1 diabetes risk were also associated with the relevant drug target genetic proxies and used Mendelian randomisation to evaluate the direction and magnitude of the associations. Furthermore, we performed Mendelian randomisation analysis restricted to functional variants within the drug target genes.

**Results:**

Co-localisation revealed that the blood expression levels of *IL2RA* (encoding IL-2 receptor subunit α [IL2RA]), *IL6R* (encoding IL-6 receptor [IL6R]) and *IL6ST* (encoding IL-6 cytokine family signal transducer [IL6ST]) shared the same causal variant with type 1 diabetes liability near the corresponding genes (posterior probabilities 100%, 96.5% and 97.0%, respectively). The OR (95% CI) of type 1 diabetes per 1-SD increase in the genetically proxied gene expression of *IL2RA*, *IL6R* and *IL6ST* were 0.22 (0.17, 0.27), 1.98 (1.48, 2.65) and 1.90 (1.45, 2.48), respectively. Using missense variants, genetically proxied *TYK2* (encoding tyrosine kinase 2) expression levels were associated with type 1 diabetes risk (OR 0.61 [95% CI 0.54, 0.69]).

**Conclusions/interpretation:**

Our findings support the targeting of IL-2, IL-6 and TYK2 signalling in prevention of type 1 diabetes.

**Data availability:**

The analysis code is available at https://github.com/jkoskenniemi/T1DSCREEN, which also includes instructions on how to download the original GWAS summary statistics.

**Graphical Abstract:**

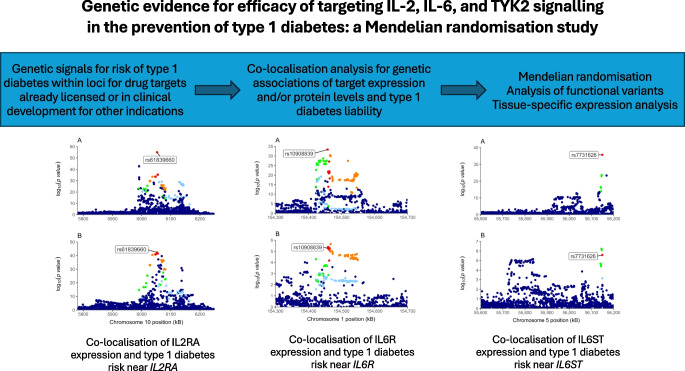

**Supplementary Information:**

The online version of this article (10.1007/s00125-024-06267-5) contains peer-reviewed but unedited supplementary material.


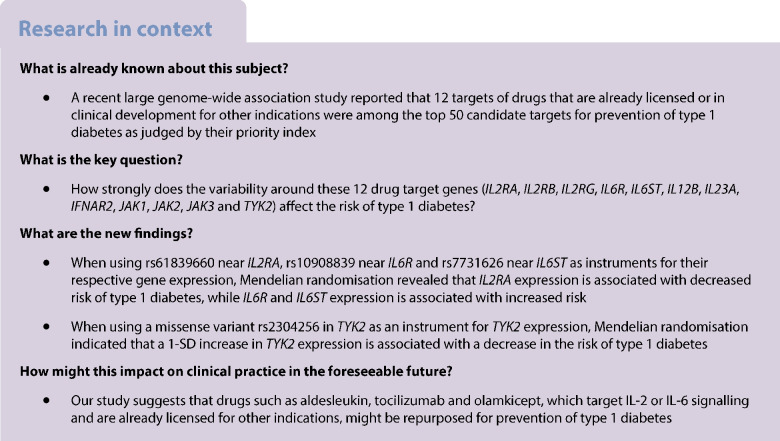


## Introduction

Type 1 diabetes is an autoimmune disease characterised by the loss of beta cell function. Despite advances in continuous glucose monitoring and insulin administration, managing type 1 diabetes remains a significant burden and few individuals reach glycaemic targets [1]. Several drugs have shown potential in delaying the loss in beta cell function in newly diagnosed diabetes [2] and teplizumab, a monoclonal anti-CD3 antibody, even delayed the onset of the clinical disease by a median of 2 years [3]. However, no current therapy can completely halt the disease progression and therefore it is crucial to identify new efficacious drug targets.

Drug targets backed by genetic evidence have higher success rates in clinical development [4]. Genome-wide association studies (GWAS) enable the discovery of genomic regions strongly associated with the disease of interest. These associations can be considered as evidence for the involvement of the corresponding proteins in the disease pathogenesis, implying that these proteins are potential drug targets for the disease. The evidence for a likely drug target can be further investigated via co-localisation and Mendelian randomisation. Co-localisation can be used to study whether the same causal variant is shared between the drug target and the disease liability or whether the loci of the risk allele and an allele influencing the drug target are distinct [5]. Furthermore, Mendelian randomisation can be used to assess how much the genetic variability in drug target levels affects the risk of a disease in the population [6].

To inform the prioritisation of targets for prevention of type 1 diabetes, we aimed to investigate the genetic evidence for the efficacy of 12 drug targets in prevention of type 1 diabetes. These targets were selected because a prior GWAS reported that they are associated with the risk of type 1 diabetes and drugs that target them are already licensed for indications other than type 1 diabetes or have progressed to clinical development (see electronic supplementary material [ESM] Table 1).

## Methods

### Study design

We selected the drug targets based on a previous GWAS of type 1 diabetes by Robertson et al [7] (Table 1 and ESM Fig. 1). Using a priority index, we ranked the drugs based on the following four factors: (1) existence of genetic variant(s) close to the potential target; (2) chromatin accessibility; (3) gene expression data in relevant cell types; and (4) protein–protein interactions [8]. We focused on 12 proteins (IL-2 receptor subunit α [IL2RA], IL-2 receptor subunit β [IL2RB], IL-2 receptor subunit γ [IL2RG], IL-6 receptor [IL6R], IL-6 cytokine family signal transducer [IL6ST], IL-12 subunit β [IL12B], IL-23 subunit α [IL23A], IFN-α and -β receptor subunit 2 [IFNAR2], Janus kinase 1 [JAK1], Janus kinase 2 [JAK2], Janus kinase 3 [JAK3] and tyrosine kinase 2 [TYK2]) that have already been targeted in clinical trials for autoimmune diseases and some of which have been licensed for indications other than type 1 diabetes (ESM Table 1) [7]. Among these targets, eight target genes (*IL2RA*, *IL2RB*, *IL6R*, *IL6ST*, *IL23A*, *JAK2*, *JAK3*, *TYK2*) had a locus associated with the risk of type 1 diabetes (*p*<1 × 10^−5^) within a distance of 1 million base pairs from the target gene (see ESM Fig. 2). *IL23A* was excluded from the analyses because data on its circulating levels were unavailable, leaving seven targets for the subsequent analyses. Genomic regions under investigation are listed in Table 1.

**Table 1:** Genomic regions under investigation

Gene	Chromosome	Start position^a^	End position^a^	Variants available^b^
*IL2RA*	10	6,010,689	6,062,367	9683
*IL2RB*	22	37,118,666	37,175,118	5489
*IL23A*	12	56,338,884	56,340,410	No pQTL data available
*IL6R*^c^	1	154,405,193	154,469,450	pQTL:8116 eQTL: 4274
*IL6ST*^c^	5	55,935,095	55,995,022	pQTL:12,024 eQTL:4253
*JAK2*	9	4,984,390	5,129,948	7939
*JAK3*	19	17,824,780	17,848,071	7142
*TYK2*	19	10,350,533	10,380,608	6403

^a^Using genome build hg38

^b^Within ±1 million base pairs from the gene, referring to eQTL data if not stated otherwise

^c^Both pQTL and eQTL data were analysed because soluble and membrane-bound IL-6R and gp130 (encoded by *IL6ST*) have different biological actions (see ESM Fig. 3)

All primary studies that generated the GWAS summary statistics used in our analysis have undergone institutional board review, have received ethical approval and were conducted according to declaration of Helsinki [9–11].

### Participants

We obtained the GWAS summary statistics for type 1 diabetes, whole-blood gene expression (expression quantitative trait loci [eQTL]) and serum protein levels (protein quantitative trait loci [pQTL]) (ESM Table 2). eQTL data were used for loci near the drug target genes since they encode intracellular or membrane-bound proteins. However, pQTL data were also analysed for loci in the vicinity of *IL6ST* and *IL6R* genes, since soluble forms of their proteins (gp130 and IL-6 receptor [IL-6R], respectively) may also modulate IL-6 signalling (see ESM Fig. 3) [12]. We included loci within 1 million base pairs of the seven potential drug target genes (Table 1). We obtained the data on type 1 diabetes risk variants from a subsequent GWAS of 18,942 cases and 501,638 controls of European ancestry from nine cohorts [9]. We obtained pQTL data for *IL6ST* from a GWAS of 35,559 Icelanders and eQTL data from GWAS of 31,684 individuals from 37 eQTLGen Consortium cohorts, most individuals being European [10, 11]. We summarise the study population details of the utilised GWAS, as well as methods of ascertainment of cases of type 1 diabetes, measurement of whole-blood gene expression and serum protein levels, in ESM Methods.

### Co-localisation

We conducted co-localisation analysis to assess whether the genetic associations for type 1 diabetes risk near the seven drug target genes align with those for the whole-blood gene expression or serum protein levels of these targets. We performed a co-localisation analysis using a ‘coloc’ package in R [5]. This method uses Bayesian principles to assess the relationship between two traits. It considers all variants within a specific genetic locus and evaluates the following hypotheses (H0–H4), assuming a maximum of one causal variant per trait:

- H0: there is no association with either trait, implying no specific causal variants
- H1: there is an association with the exposure trait only
- H2: there is an association with the outcome trait only
- H3: there are associations with both exposure and outcome traits, two independent SNPs (i.e. distinct casual variants)
- H4: there are associations with both exposure and outcome traits, one shared causal variant

A high posterior probability for H4 implies a shared causal variant for the two traits. A substantial posterior probability for H3 suggests the two traits are influenced by distinct causal variants linked to each trait. We used the default prior probabilities of 1 × 10^−4^, 1 × 10^−4^ and 1 × 10^−5^ for a variant being associated with the exposure trait, the outcome trait and both traits, respectively.

### Mendelian randomisation

To evaluate the direction and magnitude of the causal effects, we performed Mendelian randomisation for those variants that co-localised between drug target levels and the risk of type 1 diabetes (posterior probability for H4 >0.8). Mendelian randomisation uses genetic variants to investigate the relationship between an exposure (e.g. drug target levels) and an outcome (e.g. type 1 diabetes risk) for causality. Under Mendel’s law of assortment, genetic variants are accepted to be independent of other genetic alleles and can be used as valid instrumental variables to estimate the causal effect of the exposure on the outcome if the following three assumptions are fulfilled [6].

1. the genetic variant is associated with the exposure
2. the genetic variant is associated with the outcome only through the exposure
3. the genetic variant is not associated with any confounders

Advantages of Mendelian randomisation include limited susceptibility to reverse causation and confounding by external factors that influence both exposure and the outcome. Linkage disequilibrium (LD) may confound Mendelian randomisation if genetic variants are not shared between the exposure and outcome but they reside in the same genomic area. However, we investigated this possibility in the prior co-localisation step.

### Examination of functional variants near drug target genes

To further examine the potential causality of the putative drug targets on type 1 diabetes risk, we searched for functional missense variants in the coding area of these seven drug target genes from PhenoScanner [13] that were associated with the protein/expression levels of the target at *p*<1 × 10^−5^. We sought for missense variants only within the gene region for each gene. We used a more lenient threshold than the genome-wide significance of *p*<5 × 10^−8^ since we only focused on *cis*-variants, and the threshold used can be interpreted as a Bonferroni-corrected threshold for 5000 independent variants. These variants were then individually used as instruments in Mendelian randomisation to test for causality of the targets on the risk of type 1 diabetes.

### Tissue-specific gene expression analyses using bulk tissue and single-cell eQTL data

To further assess the tissue specificity of the regions that showed evidence for association in Mendelian randomisation, we conducted co-localisation with tissue-specific gene expression and type 1 diabetes liability. We obtained data for *IL2RA*, *IL6R*, *IL6ST* and *TYK2* single-cell eQTL of data for immune cell subsets from a GWAS of 982 individuals from the OneK1K cohort, and spleen and pancreas eQTL data from the Genotype Tissue Expression (GTEx) project (v8) [14, 15].

### Statistical analysis

All analyses were done with R version 4.2 (R Foundation for Statistical Computing, Vienna, Austria) using the ‘coloc’ and ‘TwoSampleMR’ packages [5, 16]. Effect size for change in the risk of type 1 diabetes is reported per change in SD of serum protein or mRNA levels, and the genetic variants were not weighted in any of the analyses. β and SE values for whole-blood RNA levels were calculated using formulae $\beta ={z\left(\sqrt{2p\left(1-p\right)\left(n+{z}^{2}\right)}\right)}^{-1}$ and $SE={2p\left(1-p\right)\left(n+{z}^{2}\right)}^{-1}$, where *p* = minor allele frequency, *n* = sample size, and *z* = *z* score. Mendelian randomisation estimates are reported as Wald estimates. To assess the instrument strength and potential weak instrument bias, we calculated the *F* statistics for the instruments using formula *F* = (β/SE)^2^.

We only used variants that were available in the GWAS summary statistics for both exposure and outcome traits within each genomic locus. All the LD *r*^2^ values reported in this study were obtained from the European population of the 1000 Genomes project using the package ‘ieugwasr’. The study protocol was not preregistered for this study. The code used to generate our results is available at https://github.com/jkoskenniemi/T1DSCREEN.

## Results

### The same genetic variants affect type 1 diabetes risk and whole-blood *IL2RA*, *IL6R* and *IL6ST* gene expression

**Fig. 1:**
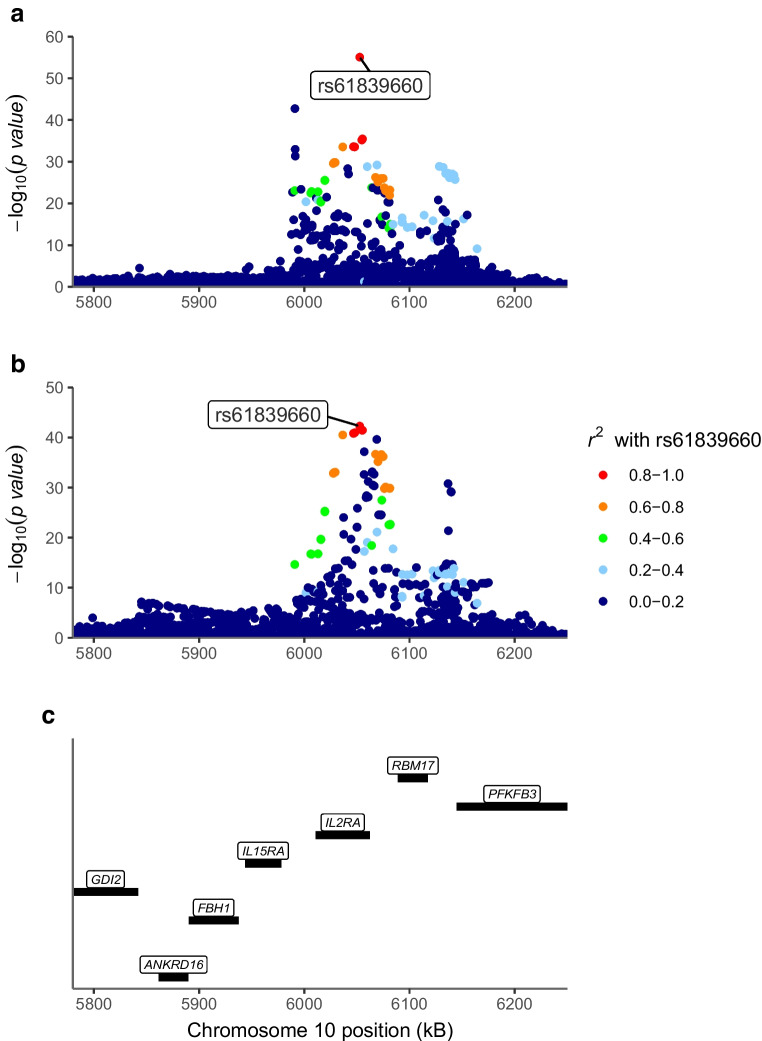
Regional Manhattan plot of whole-blood IL2RA expression (**a**) and risk of type 1 diabetes (**b**) near *IL2RA* (**c**). The colours indicate the LD *r*^*2*^ value with rs61839660 (the most likely shared causal variant identified in co-localisation), based on 1000Genomes European reference

**Fig. 2:**
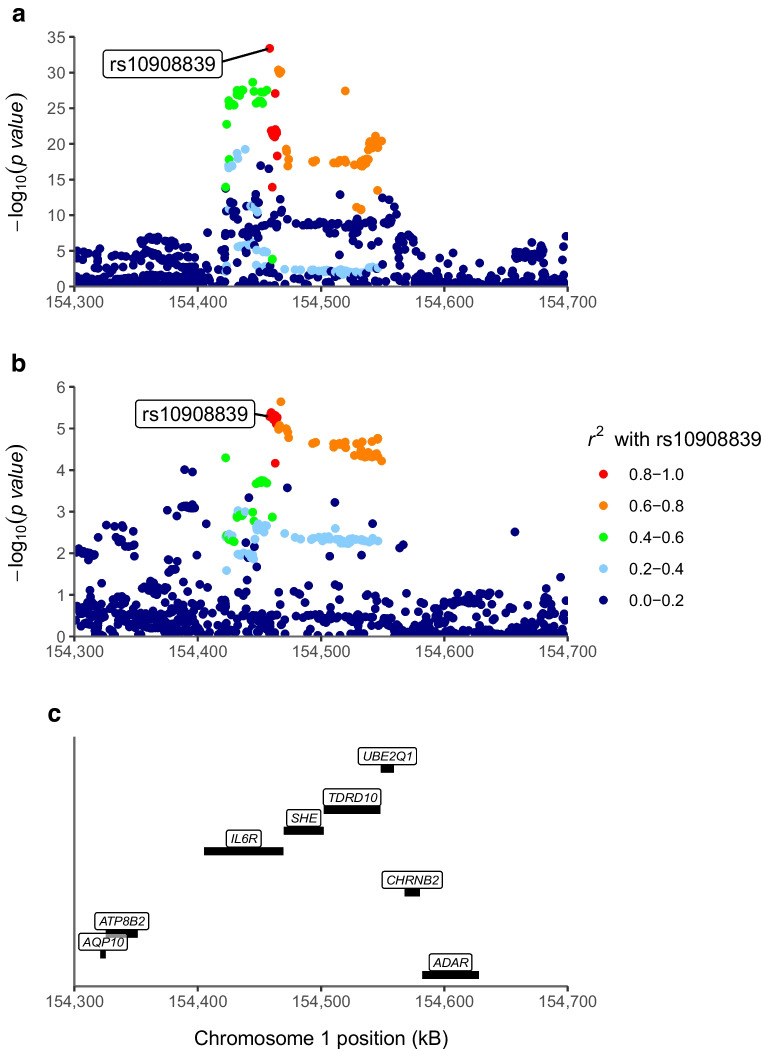
Regional Manhattan plot of whole-blood IL6R expression (**a**) and risk of type 1 diabetes (**b**) near *IL6R* (**c**). The colours indicate the LD *r*^*2*^ value with rs10908839 (the most likely shared causal variant identified in co-localisation), based on 1000Genomes European reference

We found evidence for a shared causal variant between the risk of type 1 diabetes and whole-blood *IL2RA* (rs61839660, posterior probability 100%, Fig. 1), *IL6R* (rs10908839, posterior probability 96.5%, Fig. 2) and *IL6ST* gene expression (rs7731626, posterior probability 97.0%, Table 2 and ESM Fig. 4). The variant rs10908839 is in strong LD (*r*^2^=0.76) with the previously identified lead SNP rs2229238 associated with the risk of type 1 diabetes in the study by Robertson et al [7]. No evidence for co-localisation was observed between drug target levels and the risk of type 1 diabetes near the other target genes (Table 2 and ESM Figs 5–10).

**Table 2:** Results of co-localisation between drug target (eQTL/pQTL) and risk of type 1 diabetes

Gene	Trait	H0	H1	H2	H3	H4
*IL2RA*	eQTL	0.0	0.0	0.0	0.0	100.0
*IL2RB*	eQTL	0.0	0.0	0.0	99.7	0.3
*IL6R*	eQTL	0.0	0.5	0.0	2.9	96.5
*IL6R*	pQTL	0.0	46.1	0.0	51.7	2.2
*IL6ST*	eQTL	0.0	0.3	0.0	2.8	97.0
*IL6ST*	pQTL	0.0	9.3	0.0	90.5	0.2
*JAK2*	eQTL	0.0	0.0	0.0	100.0	0.0
*JAK3*	eQTL	0.0	1.2	0.3	98.5	0.0
*TYK2*	eQTL	0.0	0.0	0.0	86.6	13.4

Data are presented as posterior probabilities (%)

H0, no association with either trait; H1, an association with the exposure trait only; H2, an association with the outcome trait only; H3, associations with both exposure and outcome traits, two independent SNPs (i.e. distinct causal variants); H4, associations with both exposure and outcome traits, one shared causal variant

### Genetically proxied variability in *IL2RA*, *IL6R* and *IL6ST* expression is associated with the risk of type 1 diabetes

We investigated, using Mendelian randomisation, the direction and magnitude of the expected change in the risk of type 1 diabetes if *IL2RA*, *IL6R* and *IL6ST* were targeted, using variants identified as the most likely shared causal variant in co-localisation (rs61839660 for *IL2RA* gene expression, *F*=248; variant rs10908839 for *IL6R* gene expression, *F*=148; and variant rs7731626 for IL6ST expression, *F*=159). The results revealed an OR of 0.22 (95% CI 0.17, 0.27) for type 1 diabetes risk (*p*=5.3 × 10^−43^) per SD increase in genetically proxied IL2RA expression, an OR of 1.98 (95% CI 1.48, 2.65) per SD increase in genetically proxied IL6R expression (*p*=5.2 × 10^−6^) and an OR of 1.90 (95% CI 1.45, 2.48) per SD increase in genetically proxied IL6ST expression (*p*=2.6 × 10^−6^; Table 3).

**Table 3:** Mendelian randomisation results for IL2RA, IL6R, IL6ST, JAK2 and TYK2

Target	Lead variant identified in co-localisation	Mendelian randomisation OR (95% CI)	*p* value	Lead functional variant	Mendelian randomisation OR (95% CI)	*p* value
*IL2RA*	rs61839660	0.22 (0.17, 0.27)	5.3 × 10^−43^	–	–	–
*IL6R*	rs10908839	1.98 (1.48, 2.65)	5.2 × 10^−6^	–	–	–
*IL6ST*	rs7731626	1.90 (1.45, 2.48)	2.6 × 10^−6^	rs141500365	0.98 (0.78, 1.20)	0.84
*JAK2*	–	–	–	rs41316003	0.74 (0.46, 1.20)	0.22
*TYK2*	–	–	–	rs2304256	0.61 (0.54, 0.69)	1.4 × 10^−14^

### Functional variant in coding area of *TYK2* is associated with the risk of type 1 diabetes

We found 22 missense mutations in the examined drug target genes with GWAS summary data on type 1 diabetes risk (ESM Table 3). Most of the mutations had an allele frequency of <0.01 with the notable exceptions of rs2228145 (minor allele frequency [MAF] **=**0.39) in *IL6R* as well as rs12720356 (MAF=0.09) and rs2304256 (MAF=0.28) in *TYK2*. Summary statistics for both drug target level and type 1 diabetes risk were available for nine variants. One independent variant at LD *r*^2^<0.2 within each locus (rs41316003 in *JAK2*, rs2304256 in *TYK2* and rs141500365 *IL6ST*) was associated with the relevant drug target (protein or gene expression levels at *p*<1 × 10^−5^). When using these variants as instruments in Mendelian randomisation, the genetically proxied TYK2 expression was associated with the risk of type 1 diabetes (rs2304256: OR 0.61 [95% CI 0.54, 0.69]) whereas we found no clear evidence for association when using missense variants in *IL6ST* (OR 0.98 [95% CI 0.78, 1.20]) or *JAK2* (OR 0.74 [95% CI 0.46, 1.20]; Table 3). ESM Table 4 shows the LD matrix between the functional variants in the coding area of *TYK2* as well as the lead SNPs associated with TYK2 expression and the risk of type 1 diabetes in the vicinity of the *TYK2* gene.

### Type 1 diabetes and *IL2RA* co-localise in CD8^+^ effector memory T cells and type 1 diabetes and *IL6ST* in CD4^+^ and CD8^+^ naive and central memory T cells

To study which specific blood immune cells or tissues mediate the association between IL2RA, IL6R, IL6ST and TYK2 expression and type 1 diabetes risk, we analysed their pairwise co-localisation with type 1 diabetes risk in spleen, pancreas and subsets of blood immune cells. IL2RA expression in CD8^+^ effector memory T cells co-localised (posterior probability 99.4%, ESM Table 5) with type 1 diabetes risk, with the lead causal variant rs61839660 being the same as for whole-blood *IL2RA* mRNA expression. Neither IL6R nor TYK2 expression co-localised in any of available immune cells. However, IL6ST expression co-localised in CD4^+^ and CD8^+^ naive and central memory T cells (lead causal variant rs7731626; posterior probabilities 97.0% and 92.4%, respectively, ESM Table 5). We did not observe such robust evidence for co-localisation between *IL2RA*,* IL6R*, *IL6ST* or *TYK2* eQTL and the risk of type 1 diabetes in other cell types (ESM Table 5) or in spleen or pancreas (ESM Table 6).

## Discussion

Leveraging data from large-scale GWAS and multiple quantitative trait locus datasets, we investigated the genetic evidence for the efficacy of seven candidate drugs in prevention of type 1 diabetes. Using co-localisation and Mendelian randomisation, we found genetic evidence to support the role of IL-2 and IL-6 signalling in the pathogenesis of type 1 diabetes. In addition, the investigation of functional missense variants suggested that TYK2 signalling is involved in the aetiology of type 1 diabetes.

While the original GWAS of immune cell subsets did not report the eQTL of *IL2RA* in regulatory T cells (Tregs), our evidence for the protective effect of blood IL2RA expression on the risk of type 1 diabetes could be interpreted as supporting the role of Tregs as a natural protection against type 1 diabetes. A low but sufficient level of IL-2 is crucial for the survival and function of Tregs, which constitutively express IL2RA, IL2RB and IL2RG to produce α-, β- and γ-chains, respectively, required for trimeric high-affinity IL-2 receptors [17]. Naive T cells express IL2RB and IL2RG, required for the intermediate-affinity IL-2-receptors, but they express IL2RA only transiently when stimulated by antigen-presenting cells and are thus less stimulated by IL-2 when not activated. Thus, higher blood IL2RA expression could be a sign of increased quantity and function of Tregs, which maintain a level of tolerance towards self-peptides and decrease the risk of type 1 diabetes [18]. A previous small study showed that rs12722495, which is in strong LD (*r*^2^=0.89) with our lead *IL2RA* eQTL and type 1 diabetes risk locus rs61839660 near *IL2RA*, decreased IL2RA expression in Tregs as well as their sensitivity to IL-2 [19].

Alternatively, as IL-2 signalling increases the proliferation of the conventional T cells and Tregs alike, IL-2 signalling might not decrease the risk of type 1 diabetes only by increasing tolerance but also by promoting appropriate responses to pathogens. This is supported by our single-cell-level results, in which we observed evidence for co-localisation between IL2RA expression and the risk of type 1 diabetes only in CD8^+^ central memory T cells (eQTL of Tregs were not available as they were not distinguished from other T cells). In a birth cohort study of children at high genetic risk of type 1 diabetes (TEDDY study), presence of enteroviral DNA in stool was associated with the risk of islet autoimmunity and the association was stronger in persistent infections indicated by prolonged shedding of enteroviral DNA [20]. Likewise, children who developed islet autoimmunity presented longitudinal transcriptional signatures consistent with a less-robust immune response against enteroviral infections compared with matched control children [21]. Since the increased IL-2 signalling in CD8^+^ T cells during viral infections prioritises robust immune response against production of long-lived memory cells, this explanation might also explain why rs61839660 near *IL2RA* strongly co-localised between the risk of type 1 diabetes and the eQTL of *IL2RA* in effector memory T cells and CD8^+^ naive/central memory T cells.

The co-localisation of IL2RA expression in CD8^+^ effector memory T cells with type 1 diabetes risk is further supported by the previous reports of IL-2 impairment leading to CD8^+^ T cell exhaustion, potentially driven by both acute and chronic viral infections [22, 23]. Interestingly, our lead rs61839660 near *IL2RA*, which was associated with increased IL2RA expression and decreased risk of type 1 diabetes, was previously shown to be associated with higher risk of Crohn’s disease [24] and lower risk of type 1 diabetes [25]. This suggests that the optimal balance between effector and regulatory T cell function may vary between autoimmune diseases [17]. Regardless of the possible mechanism, our findings support the rationale of conducting type 1 diabetes prevention trials with low-dose IL-2.

Consistent with our finding of the protective effect of IL-2 signalling, we found that IL-6 signalling increased the risk of type 1 diabetes. The secretion of IL-6 from macrophages rapidly in response to infections and tissue damage promotes various acute phase responses [26]. IL-6 signalling occurs as classic signalling through a cell-membrane-bound IL-6R, *trans*-signalling through soluble circulating IL6R and membrane-bound gp130 (encoded by *IL6ST*) and *trans*-presentation by dendritic cells, in which IL-6 is presented to membrane-bound gp130 in T cells via dendritic cell-membrane-bound IL-6R (ESM Fig. 3) [27]. IL-6 inhibits the development and function Tregs and promotes the development of pathogenic T helper 17 (Th17) cells [28], which is inhibited by IL-2. Pronounced IL-6 signalling may alter the balance of Treg/Th17, a proposed causative factor in autoimmune diseases such as rheumatoid arthritis [28] and possibly also type 1 diabetes [29].

While, IL-6 *trans*-signalling and *trans*-presentation may be a more potent inducer of autoimmunity than classic signalling [12], our finding that blood IL6R and IL6ST expression are associated with increased risk of type 1 diabetes may be explained by any of the three signalling modalities. However, since IL6ST expression in CD4^+^ and CD8^+^ naive or central memory T cells co-localised with type 1 diabetes and IL6R expression did not, it is tempting to speculate that *trans*-signalling might be more important than classic IL-6 signalling in the pathogenesis of type 1 diabetes.

In contrast to our findings, tocilizumab (a monoclonal antibody against IL6R), which blocks all three IL-6 signalling modalities, did not significantly affect the decline in residual beta cell function in individuals with newly diagnosed type 1 diabetes in a randomised, placebo-controlled, double-blind clinical trial [30]. However, this discrepancy may be partially explained by the timing of the intervention. Genetic polymorphisms typically exert life-long influence on risk of diseases, including every stage of type 1 diabetes, whereas the intervention in the study by Greenbaum et al [30] took place after the diagnosis of type 1 diabetes, at which stage a sharp fall in beta cell function has already taken place [31].

Whole-blood TYK2 expression and the risk of type 1 diabetes did not co-localise, whereas when using the missense mutation rs2304256 in *TYK2* as an instrument in Mendelian randomisation, TYK2 expression was associated with type 1 diabetes risk. The absence of evidence for co-localisation may reflect violations of the one-causal-variant assumption. Indeed, the missense mutation rs2304256 is in very high LD (*r*^2^=1.00) with the lead *TYK2* eQTL rs34725611. Moreover, rs2304256 is only in moderate LD (*r*^2^=0.10) with rs144309607, the lead variant on type 1 diabetes liability in co-localisation, suggesting two independent signals. Of note, a known missense variant rs34536443 is not available in the eQTL data and therefore could not be used in co-localisation. Despite the one-causal-variant assumption, ‘coloc’ is relatively robust to multiple causal variants, and co-localisation methods allowing for multiple causal variants are highly sensitive to LD misspecifications in the reference panel [6]. Therefore, in the absence of an accurate LD reference, we proceeded with the ‘coloc’ method for our co-localisation while acknowledging its limitations.

Overall, our *TYK2* findings are consistent with previous studies suggesting that TYK2 signalling is associated with the risk of type 1 diabetes [32]. Promising results have been found in clinical trials targeting TYK2 in autoimmune diseases, supporting the potential of drug repurposing in type 1 diabetes [33]. Other reasons for the co-localisation discordance could be that TYK2 is not activated until IFN-α binding to IFNAR1 and *TYK2* RNA expressions are known to have low tissue and cell type specificity [34, 35].

It is important to note that Mendelian randomisation estimates are only valid if the instrumental variable associations (relevance, independence, exclusion restriction) are met. The strong associations between the assessed genetic polymorphisms and the studied exposures suggested that the genetic instruments studied were relevant for the studied exposures. The strategy of selecting instruments from within the *cis*-region of the exposure of interest is an established approach for investigating drug effects [36]. c*is*-Mendelian randomisation studies are by design less prone to horizontal pleiotropy and exposure misspecification (which may lead to violations of independence and exclusion restriction assumptions), as genetic variants typically exert the strongest influence on nearby genes and therefore most effects are secondary to reading of the nearby genes. However, restricting the instruments to *cis*-variants comes at the expense of potentially missing strong *trans*-variants that associate with the exposure. Furthermore, our co-localisation results suggest that associations between whole-blood *IL2RA* and *IL6R* expression and the risk of type 1 diabetes are unlikely to be caused by LD with a genetic variant that primarily influences the reading of other genes in the vicinity of *IL2RA* or *IL6R* loci.

Some aspects of generalisability of our results are also worth mentioning. While our study did not explicitly exclude individuals from non-European ancestries, the original GWAS studies primarily included individuals of European descent, which limits the spectrum of rare variants and the generalisability of our findings to other ancestries. Furthermore, our Mendelian randomisation estimates represent the influence of small changes in *IL2RA*, *IL6R* and *TYK2* expression during the entire life course before the diagnosis of type 1 diabetes. Therefore, these effect sizes cannot be directly extrapolated to clinical trials in which the doses are larger and exposures shorter, and possibly outside a key sensitive window for disease development. Thus, natural history studies and clinical prevention trials should pinpoint the optimal stage of pathogenesis at which to interfere with IL-2, IL-6 or TYK2 signalling to prevent type 1 diabetes. Finally, even if up to 50% of variability in genetic risk of type 1 diabetes is attributable to HLA-II locus [37], we could not analyse the interactions between SNPs reported here and the HLA genotype or genetic risk scores on risk of type 1 diabetes, or any sex-specific effects, as we did not have access to individual-level data.

In conclusion, our results provide genetic evidence that IL-2, IL-6 and TYK2 signalling are associated with type 1 diabetes risk. Our findings suggest that clinical trials investigating the efficacy of drugs such as tocilizumab (IL-6R antagonist that targets all IL-6 signalling modalities), olamkicept (soluble gp130Fc that blocks IL-6 *trans*-signalling) and low-dose aldesleukin (IL-2 analogue) may be promising candidates for the prevention of type 1 diabetes.

## Supplementary Information

Below is the link to the electronic supplementary material.

ESM (PDF 2922 KB)

## Data Availability

Analysis code can be accessed at https://github.com/jkoskenniemi/T1DSCREEN, which also includes instructions on how to download the original GWAS summary statistics.

## Abbreviations

eQTL: Expression quantitative trait locus / loci
GWAS: Genome-wide association study
IL2RA: IL-2 receptor subunit α
IL2RB: IL-2 receptor subunit β
IL2RG: IL-2 receptor subunit γ
IL-6R: IL-6 receptor
IL6ST: IL-6 cytokine family signal transducer
JAK1: Janus kinase 1
JAK2: Janus kinase 2
JAK3: Janus kinase 3
LD: Linkage disequilibrium
MAF: Minor allele frequency
NK: Natural killers
pQTL: Protein quantitative trait locus / loci
Th17: T helper 17
Treg: Regulatory T cell
TYK2: Tyrosine kinase 2
