# Supplementary material for: Genetic evidence for efficacy of targeting IL-2, IL-6 and TYK2 signalling in the prevention of type 1 diabetes: a Mendelian randomisation study
Source: Diabetologia. 2024 Sep 13;67(12):2667–77. doi: 10.1007/s00125-024-06267-5 (PMC11604673; doi:10.1007/s00125-024-06267-5)
Supplement: Supplementary file 1 — ESM (PDF 2922 KB) [file 125_2024_6267_MOESM1_ESM.pdf]

Genetic evidence for efficacy of targeting IL-2, IL-6, and TYK2 signalling in prevention of type 1 diabetes: A Mendelian randomisation study – Electronic Supplementary Material (ESM)

Contents

ESM Methods 2

ESM Fig. 1. Flow chart of the selection of the targets. 3

ESM Fig. 2. Selected genomic areas of interest in Robertson et al. [4]. 4

ESM Fig. 3. Schematic illustration of IL-6 signalling. 5

ESM Fig. 4. Manhattan plot of whole-blood IL6ST expression and risk of type 1 diabetes near *IL6ST*. 6

ESM Fig. 5. Manhattan plot of whole-blood IL2RB expression and risk of type 1 diabetes near *IL2RB*. 7

ESM Fig. 6. Manhattan plot of serum IL6R expression and risk of type 1 diabetes near *IL6R*. 8

ESM Fig. 7. Manhattan plot of serum IL6ST levels and risk of type 1 diabetes near *IL6ST*. 9

ESM Fig. 8. Manhattan plot of whole-blood JAK2 expression and risk of type 1 diabetes near *JAK2*. 10

ESM Fig. 9. Manhattan plot of whole-blood JAK3 expression and risk of type 1 diabetes near *JAK3*. 11

ESM Fig. 10. Manhattan plot of whole-blood TYK2 expression and risk of type 1 diabetes near *TYK2*. 12

ESM Table 1. Selected drug targets. 13

ESM Table 2. Source studies of GWAS summary statistics. 14

ESM Table 3. Functional variants in the examined seven drug targets. 15

ESM Table 4. Linkage disequilibrium (LD) matrix (r^2) between four genetic variants near *TYK2* gene. 16

ESM Table 5. Co-localisation results for single-cell expression levels and type 1 diabetes risk. 17

ESM Table 6. Co-localisation results for bulk tissue expression levels and type 1 diabetes risk in disease-relevant tissues. 19

References 20

ESM Methods

We obtained genome-wide association study (GWAS) summary statistics for type 1 diabetes, whole-blood gene expression (expression quantitative trait loci, eQTL) and serum protein levels (protein quantitative trait loci, pQTL). The serum protein levels were measured with 4,907 aptamers in 35,559 Icelanders using the SomaScan multiplex aptamer assay (version 4), including 4,907 relevant aptamers, which target 4,719 proteins [1]. Plasma samples were collected from 40,004 Icelanders between 2000 and 2019. A total of 52% of the participants were from the Icelandic Cancer Project (newly diagnosed cancer patients and their relatives and controls selected from the National Registry) and 48% were from genetic programs at deCODE genetics, mainly from the population-based deCODE Health study. In the Icelandic Cancer Project, participants were patients with newly diagnosed cancer and their relatives. The average age of the participants was 55 years and 57% of the participants were women [1].

The data for gene expression was obtained from 31,684 individuals through 37 eQTLGen Consortium cohorts [2]. 25,482 of the samples were from whole blood and 6,202 were peripheral blood mononuclear cell samples. The majority of samples were of European ancestry and from population-based cohorts. Gene expression levels of samples were measured by Illumina, Affymetrix, Affymetrix Hu-Ex version 1.0 ST expression arrays and RNA-seq. In a large-scale meta-analysis, cis-eQTL was identified for 16,987 genes, trans-eQTL for 6,298 genes and eQTS effects for 2,568 genes. In cis-eQTL, gene expression levels are affected by a proximal (<1Mb) SNP and in trans-eQTL by a distal (>5Mb) SNP or a SNP on another chromosome [2].

The GWAS data for the risk of type 1 diabetes is from nine cohorts including 18,942 individuals with type 1 diabetes and 501,638 control individuals of European ancestry [3]. The type 1 diabetes case cohorts were combined to the control cohorts based on genotyping array (Affymetrix, Illumina Infinium, Illumina Omni, and Immunochip) and ancestry (Ireland, UK or USA). For the GENIE-UK cohort, no matched country of origin was found for the control cohort so individuals of British ancestry from the University of Michigan Health and Retirement study (HRS) were used as controls. For the UK Biobank cohort and non-UK Biobank cohorts, non-European ancestry samples were removed. For control cohorts, phenotype files were used to remove individuals with type 2 diabetes or autoimmune diseases. ICD10 was used in the UK Biobank cohort to identify 1,445 type 1 diabetes cases (diagnosis of type 1 diabetes and insulin treatment within a year of diagnosis, no type 2 diabetes diagnosis). The 362,050 control individuals had no diagnosis of type 1 diabetes, type 2 diabetes, gestational diabetes or autoimmune disease [3].

ESM Fig. 1. Flow chart of the selection of the targets.

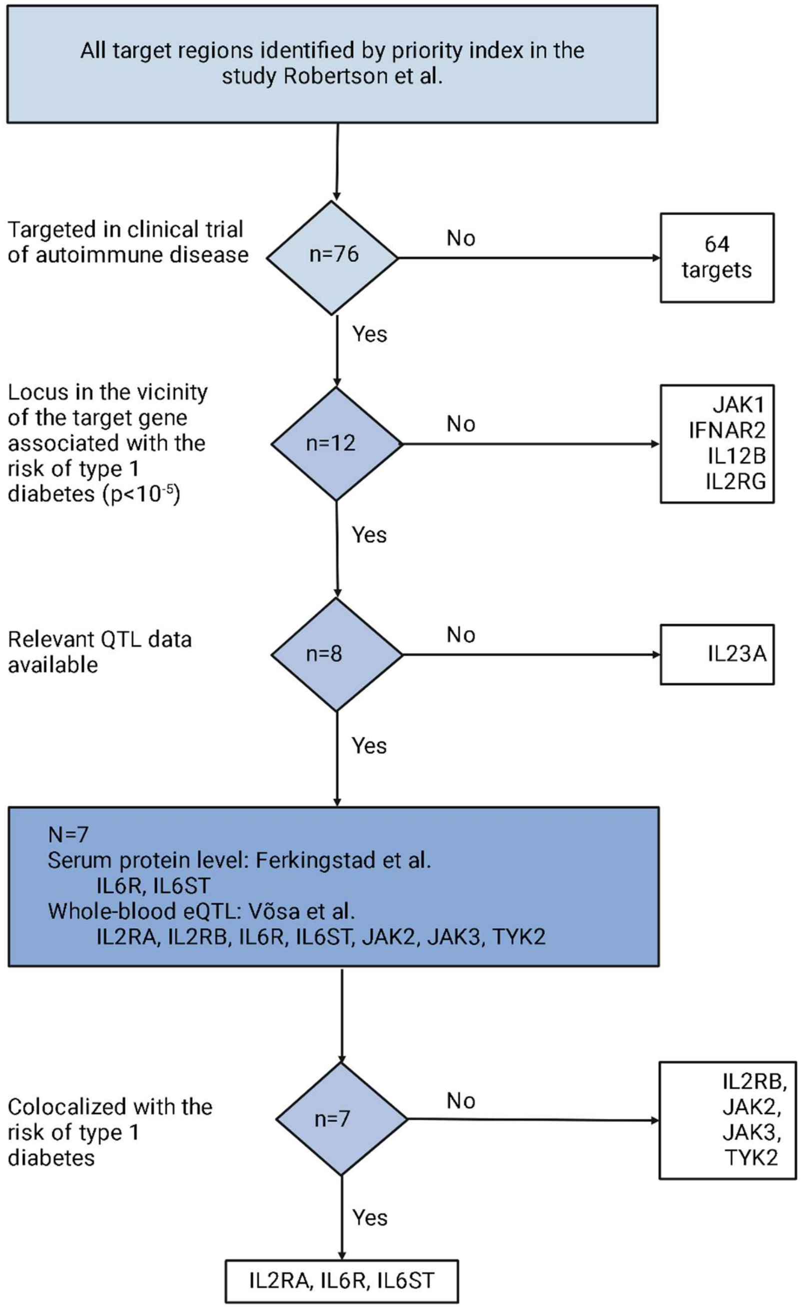

Created with BioRender.com.

ESM Fig. 2. Selected genomic areas of interest in Robertson et al. [4].

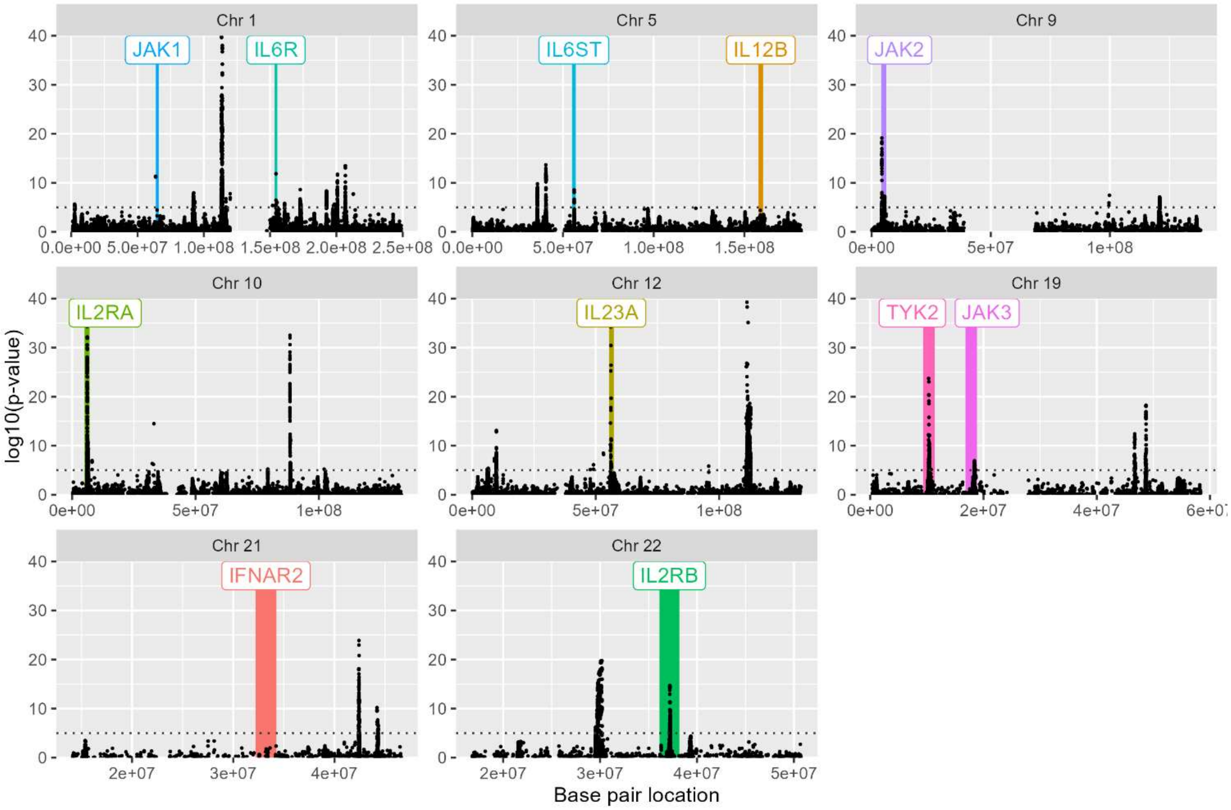

P-values < 10^{-40} are excluded from this figure. Dotted line indicates $p=10^{-5}$. Chr = Chromosome.

ESM Fig. 3. Schematic illustration of IL-6 signalling.

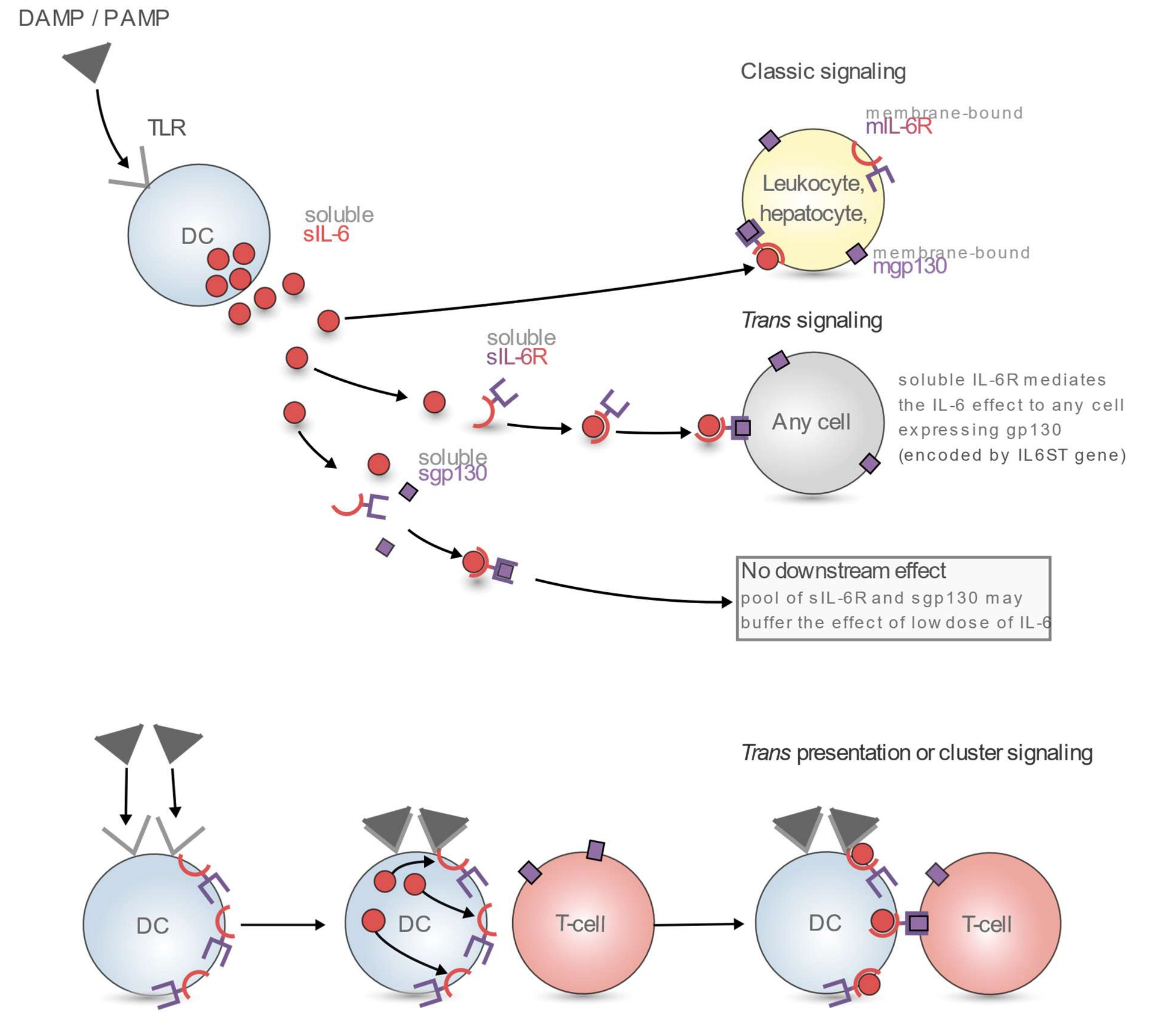

DAMP = Damage associated molecular pattern, PAMP = pathogen associated molecular pattern, TLR = Toll-like receptor, DC = dendritic cell, sIL-6 = soluble interleukin 6, mIL-6 = membrane-bound interleukin 6, sIL-6R = soluble interleukin 6 recepto mIL-6R = membrane-bound interleukin 6 receptor, sgp130 = soluble glycoprotein 130, mgp130 = membrane-bound glycoprotei 130. gp130 is encoded by IL6ST gene.

Interleukin	Effect	Genetic instrument of interleukin
soluble gp130	inhibits *trans* signaling	serum *cis*-pQTL of s-gp130 (near IL6ST)
membrane-bound gp130	increases all IL-6 signaling	whole-blood *cis*-eQTL of gp130 (near IL6ST)
soluble IL-6R	increases *trans* signaling, decreases classic signaling	serum *cis*-pQTL of s-IL-6R (near IL6R)
membrane-bound IL-6R	increases classic and *trans* signaling (possibly also *trans* presentation if in DCs)	whole blood *cis*-eQTL of IL-6R (near IL6R)

ESM Fig. 4. Manhattan plot of whole-blood IL6ST expression and risk of type 1 diabetes near *IL6ST*.

The colours indicate the LD r^2 value (based on 1000Genomes European reference) with rs7731626, the most likely shared causal variant identified in co-localisation.

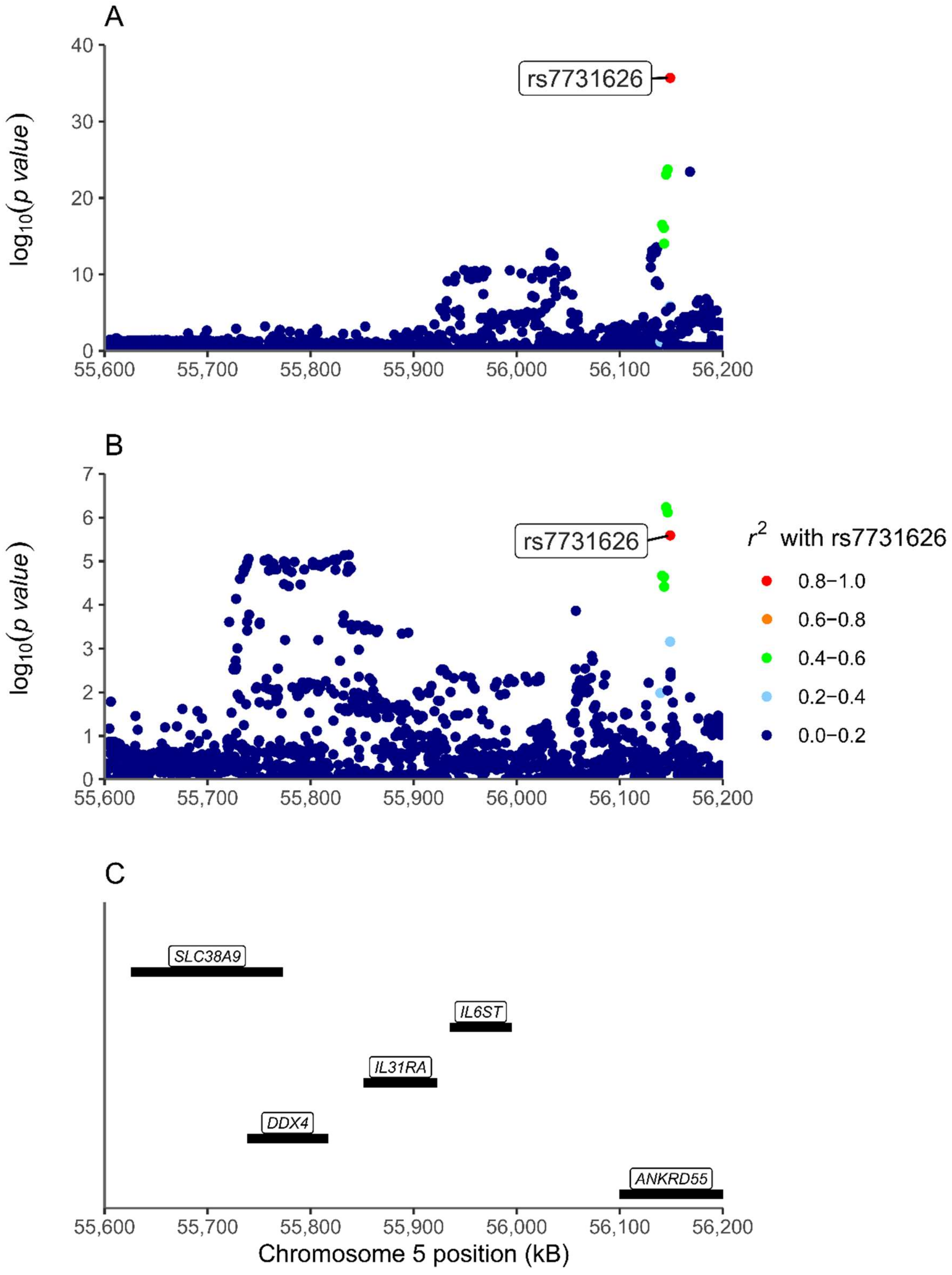

ESM Fig. 5. Manhattan plot of whole-blood IL2RB expression and risk of type 1 diabetes near *IL2RB*.

The lead variant identified in co-localisation for each trait is labelled. The colours indicate the LD r^2 value (based on 1000Genomes European reference) with rs228960.

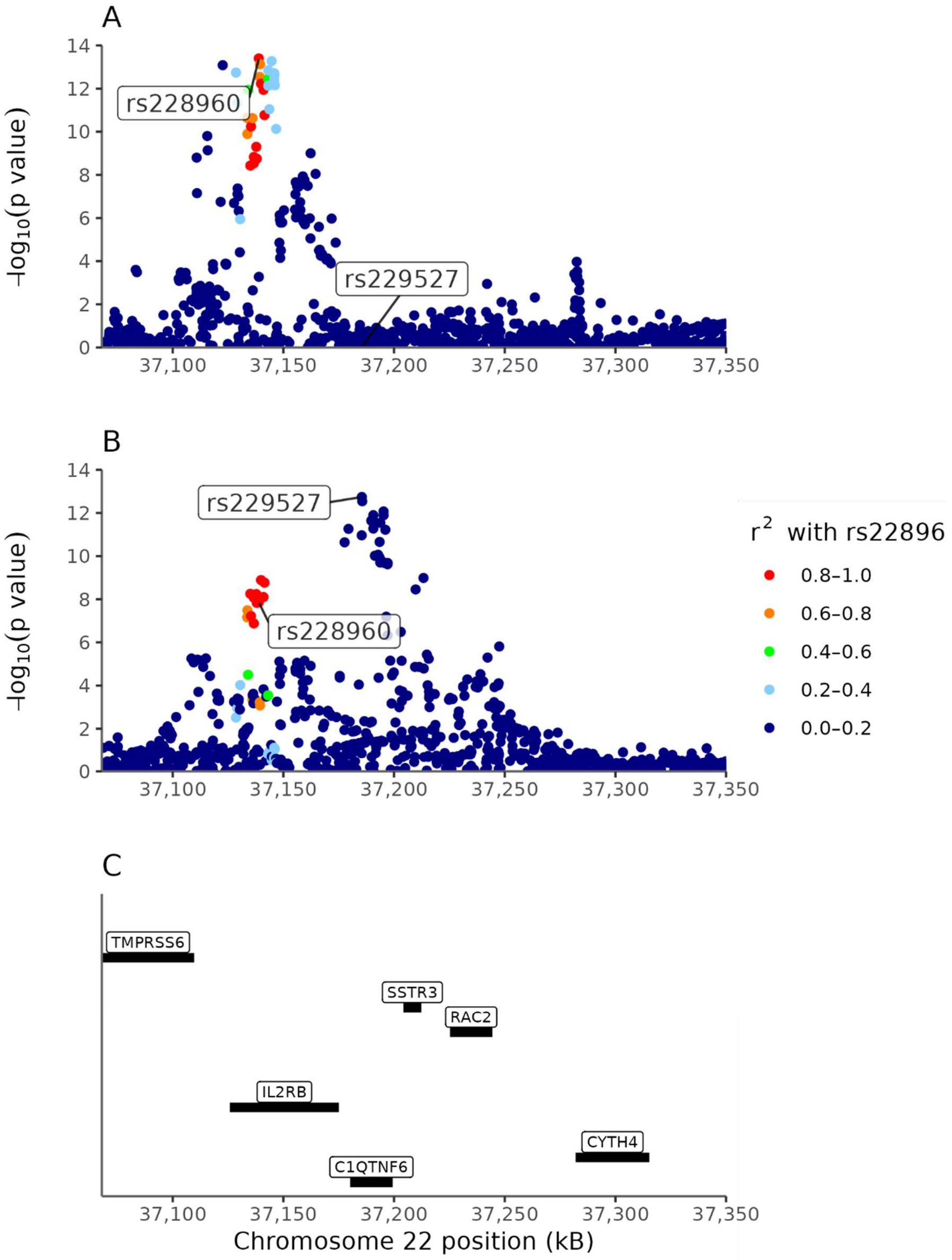

ESM Fig. 6. Manhattan plot of serum IL6R expression and risk of type 1 diabetes near *IL6R*.

The lead variant identified in co-localisation for each trait is labelled. The colours indicate the LD r^2 value (based on 1000Genomes European reference) with rs10908839.

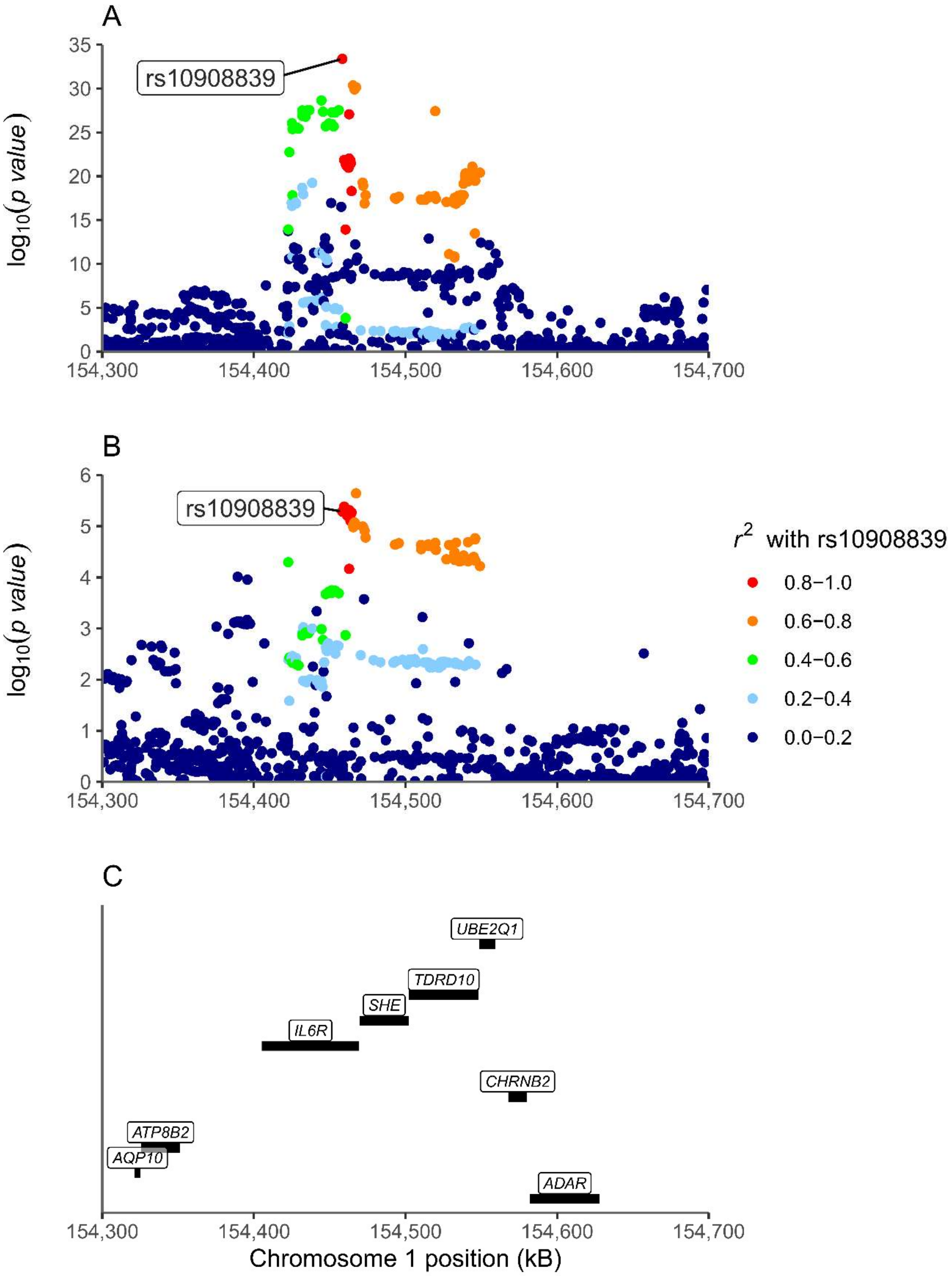

ESM Fig. 7. Manhattan plot of serum IL6ST levels and risk of type 1 diabetes near *IL6ST*.

The lead variant identified in co-localisation for each trait is labelled. The colours indicate the LD r^2 value (based on 1000Genomes European reference) with rs7730934.

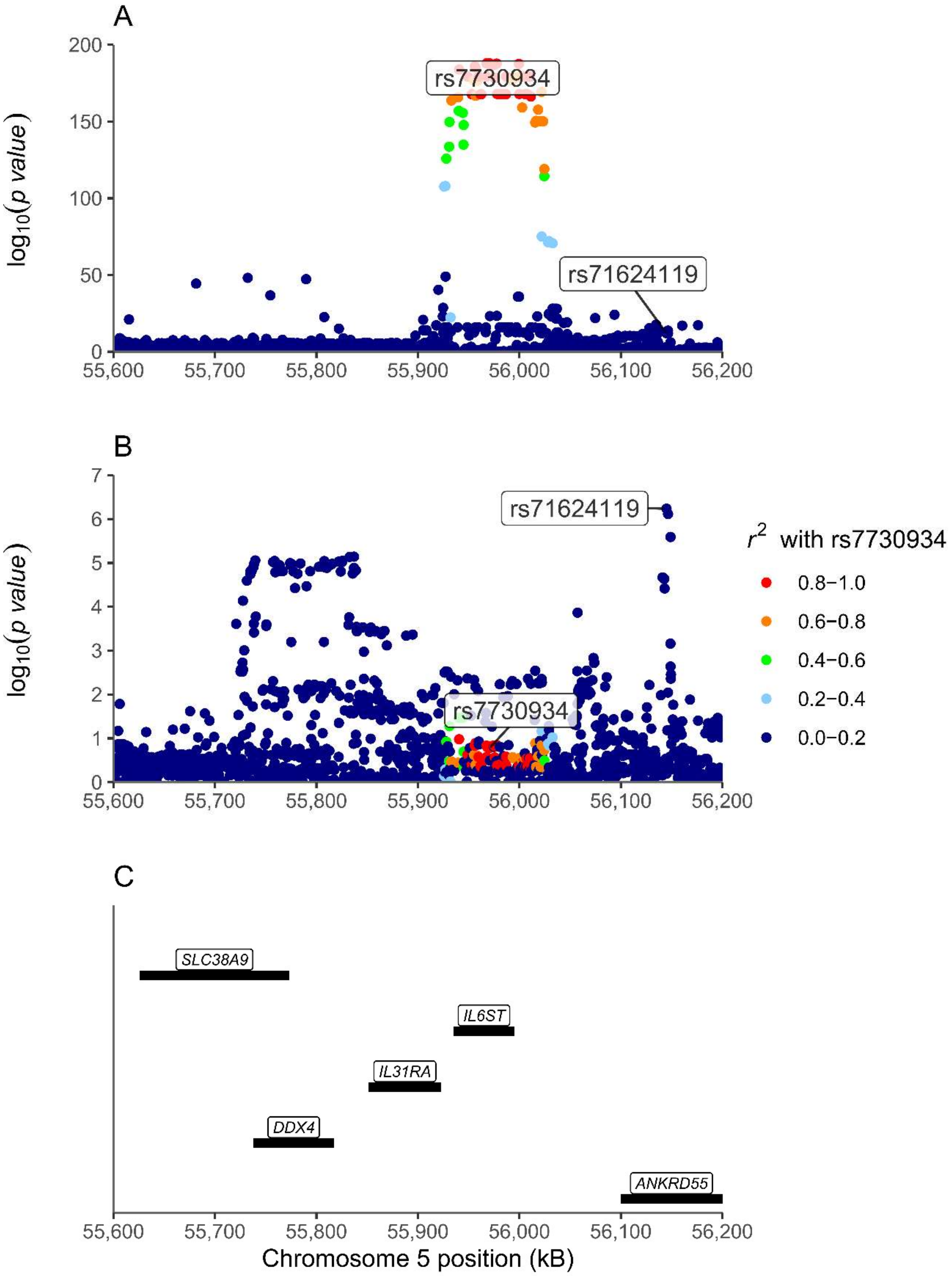

ESM Fig. 8. Manhattan plot of whole-blood JAK2 expression and risk of type 1 diabetes near *JAK2*.

The lead variant identified in co-localisation for each trait is labelled. The colours indicate the LD r^2 value (based on 1000Genomes European reference) with rs6476941.

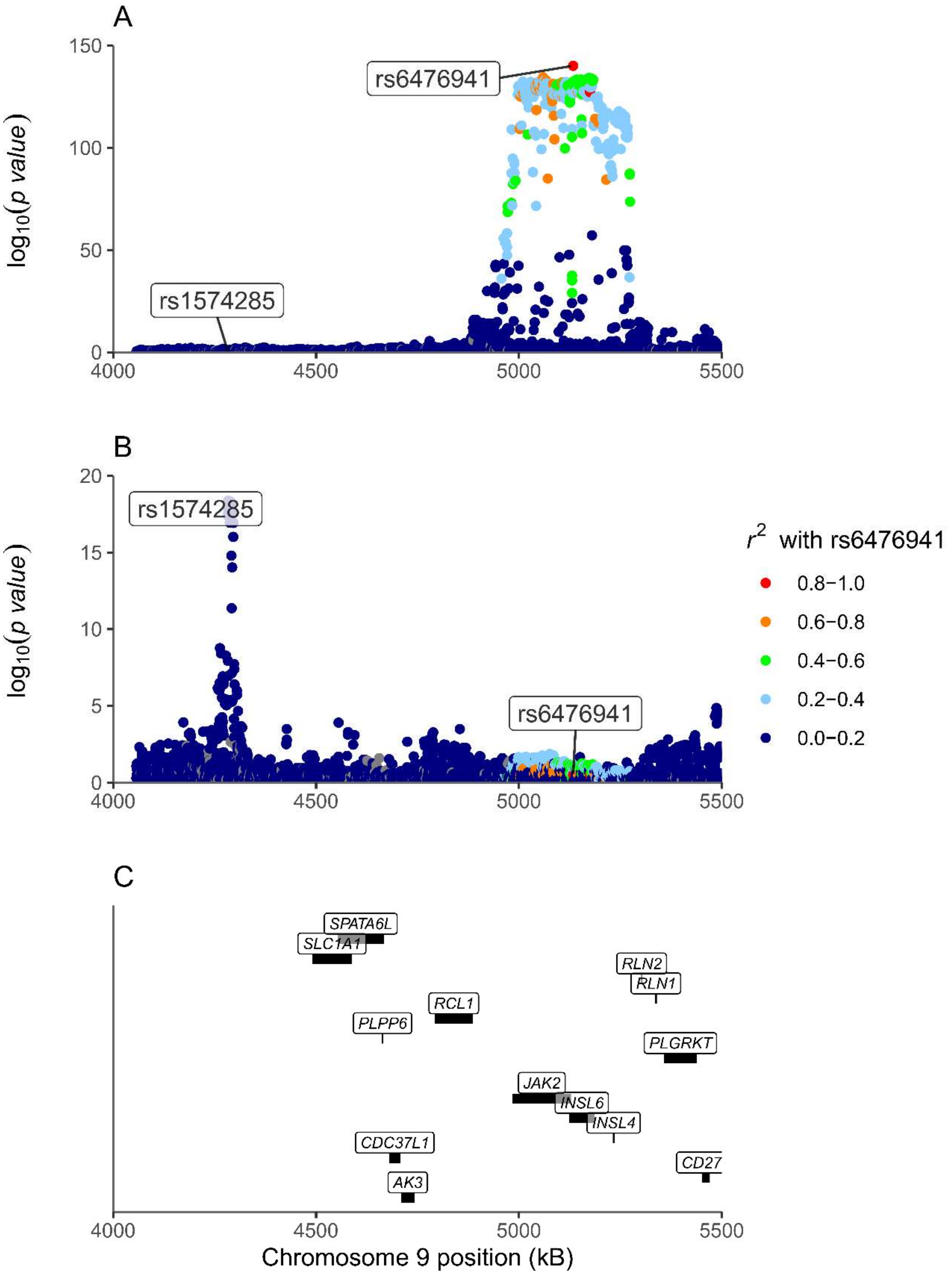

ESM Fig. 9. Manhattan plot of whole-blood JAK3 expression and risk of type 1 diabetes near *JAK3*.

The lead variant identified in co-localisation for each trait is labelled. The colours indicate the LD r^2 value (based on 1000Genomes European reference) with rs3212797.

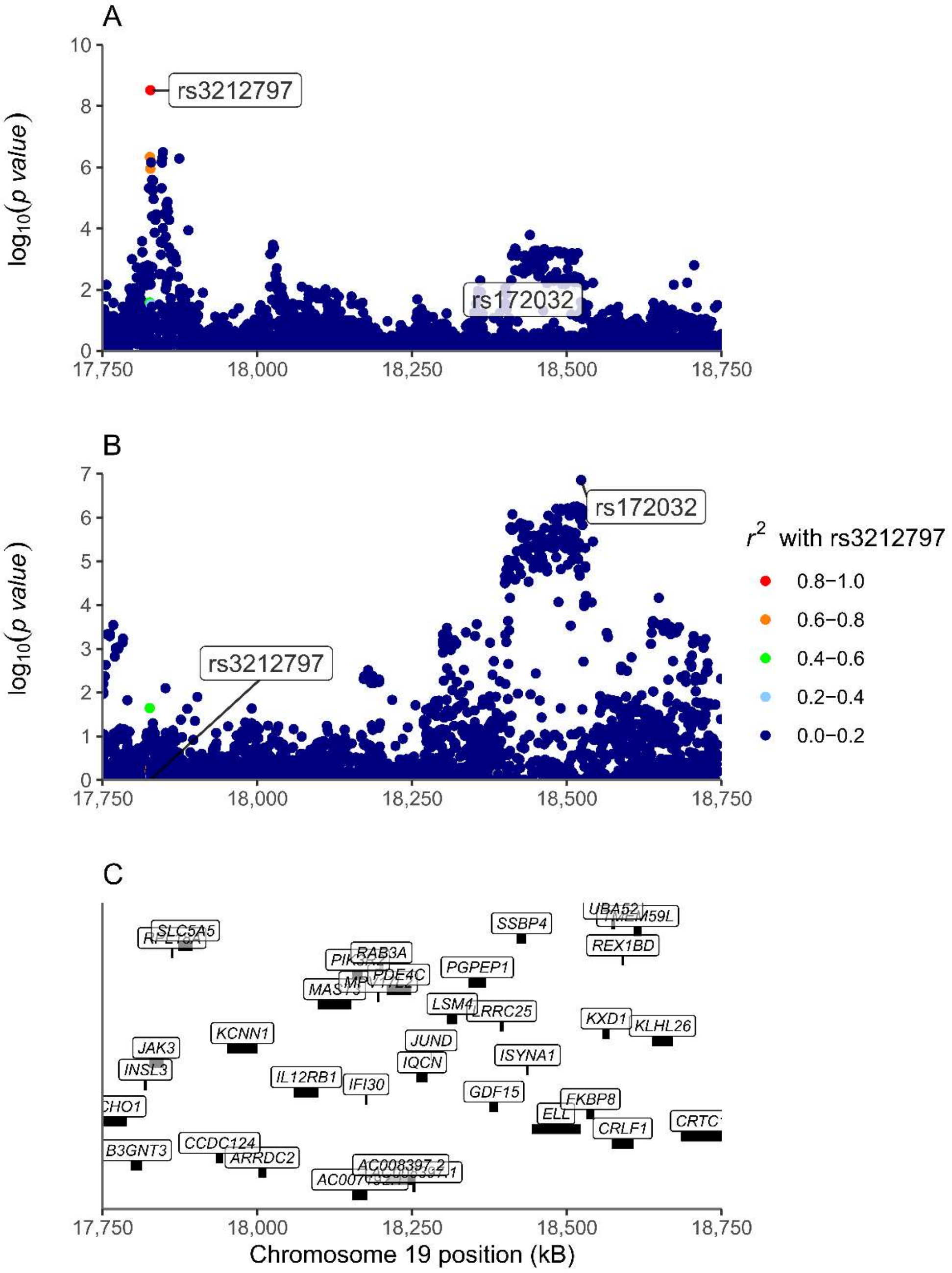

ESM Fig. 10. Manhattan plot of whole-blood TYK2 expression and risk of type 1 diabetes near *TYK2*.

The lead variant identified in co-localisation for each trait is labelled. The colours indicate the LD r^2 value (based on 1000Genomes European reference) with rs34725611.

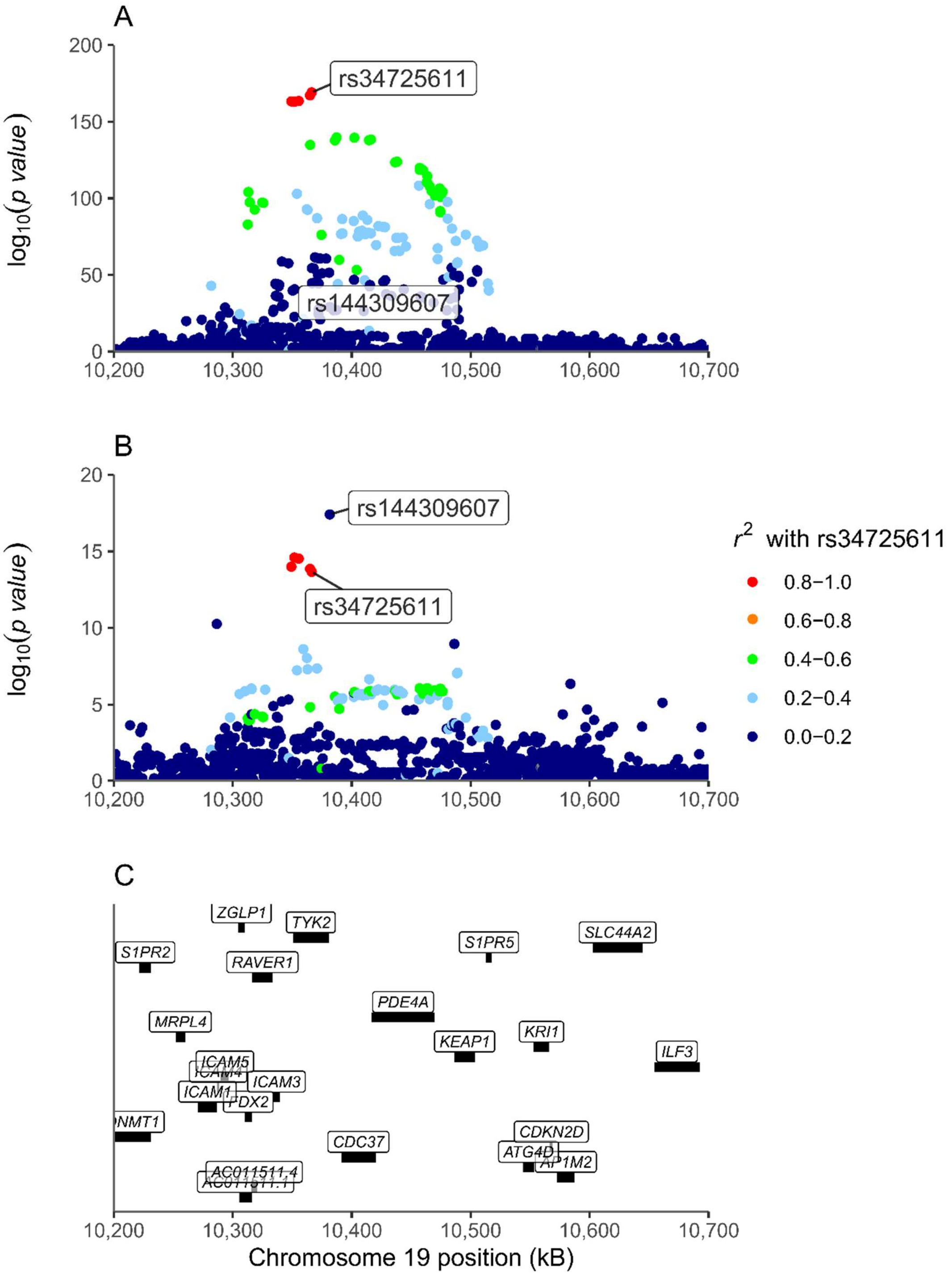

ESM Table 1. Selected drug targets.

Gene	Drug	Mechanism	Disease	Clinical status	PMID
IL2RA	Daclizumab	Anti-CD25-antibody	MS	Approved (EMA, FDA)	29661806
	Basiliximab	Anti-CD25 antibody	GVHD	Approved (EMA, FDA)	31742732
	Aldesleukin	Low dose IL-2 analog	Type 1 diabetes	Phase 2	32399500
IL2RB	Aldesleukin	Low dose IL-2 analog	Type 1 diabetes	Phase 2	32399500
IL2RG	Aldesleukin	Low dose IL-2 analog	Type 1 diabetes	Phase 2	32399500
IL6R	Tocilizumab	Anti-IL-6R antibody	RA, GCA	Approved (EMA, FDA)	28841363 34747368
	Sarilumab	Anti IL-6 and sIL-6R antibody	RA	Approved (EMA, FDA, Japan)	29931592 28290137
	Satralizumab	Anti-IL-6R antibody	NMOSD	Approved (EMA, FDA)	32797372
	Olamkicept	IL-6 and sIL-6R complex (sgp130Fc)	IBD	Phase 2a	33667488
IL6ST	Olokizumab	Anti-IL-6 ab, blocks gp130	RA	Phase 3	36001712
IL12B	Ustekinumab	IL12/23 subunit p40 blocking	UC, CD, psoriasis	Approved (EMA, FDA, AUS)	32679193 35553665
IL23A	Risankizumab	Anti-IL23A antibody	psoriasis	Approved (EMA, FDA, CAN)	28423301
	Guselkumab	Anti-IL23A antibody	psoriasis, PsA	Approved (EMA, FDA, Japan)	34460338
JAK1	Upadacitinib	JAK1 selective	RA	Approved (EMA, FDA)	31692920
	Filgotinib	JAK1 selective	RA, UC	Approved (EMA, UK, Japan)	35637376
	Tofacitinib	JAK1,3	RA	Approved (EMA, FDA)	29139090 34349573
	Abrocitinib	JAK1 selective	Atopic dermatitis	Approved (EMA, FDA)	35587593
	Baricitinib	JAK1,2	RA, atopic dermatitis	Approved (EMA, FDA)	34828623 33456084
JAK2	Fedratinib	JAK2 selective	myelofibrosis	Approved (EMA, FDA)	31571162
	Baricitinib	JAK1,2	RA, atopic dermatitis	Approved (EMA, FDA)	33456084 33240390
	Ruxolitinib	JAK1,2	myelofibrosis	Approved (EMA, FDA)	34437922
JAK3	Peficitinib	JAK1,2,3	RA	Approved (Japan)	31093950
	Decernotinib	JAK3 selective	RA	No approval	32635659
	Tofacitinib	JAK1,3	RA	Approved (EMA, FDA)	30183607
TYK2	Deucravacitinib	TYK2 selective	Psoriasis	Approved (EMA, FDA)	37168876 37168605
	Brepocitinib	TYK2/JAK1	PsA	Phase 2b	37194394
	Ropsacitinib	TYK2/JAK2 inhibitor	psoriasis, PsA	Phase 2	36592300

MS = multiple sclerosis, GVHD = graft versus host disease, RA = rheumatoid arthritis, GCA = giant cell arteritis NMOSD = Neuromyelitis optica spectrum disorders, UC = ulcerative colitis, CD = Crohns disease, IBD = inflammatory bowel disease PsA = active psoriatic arthritis. EMA = European Medicines Agency. FDA = Food and Drug Administration. The information for the drug targets featured in this table has been acquired from Drugbank Online (https://go.drugbank.com/).

ESM Table 2. Source studies of GWAS summary statistics.

Trait	Study	DOI	N
Risk of type 1 diabetes	Chiou et al. 2021	10.1038/s41586-021-03552-w	18,942 cases, 501,638 controls
Serum protein levels (except IL23A)	Ferkingstad et al. 2021	10.1038/s41588-021-00978-w	35,559
Whole blood gene expression data	Võsa et al. 2021	10.1038/s41588-021-00913-z	31,684

ESM Table 3. Functional variants in the examined seven drug targets.

	SNP	MAF	EA	NEA	β_{T1D}	SE_{T1D}	p_{T1D}	β_{QTL}	p_{QTL}	F statistic
IL2RA	rs72650666	0.004	A	G	0.18	0.15	0.22			
IL2RB	rs149508414	0.006	T	C	-0.27	0.11	0.02			
	rs143857582	0.002	T	C	-0.094	0.19	0.62			
IL6R	rs2228145	0.39	C	A	-0.044	0.014	0.002			
IL6ST	rs34417936	0.02	T	C	-0.11	0.049	0.03	-0.11	5.4×10^{-4}	
	rs61748224	0.003	T	C	0.058	0.14	0.68	-0.34	7.8×10^{-4}	
	rs141500365	0.002	A	G	0.044	0.22	0.84	-1.9	4.7×10^{-138}	630
	rs61755738	0.004	C	A	-0.015	0.11	0.89	-0.008	0.91	
JAK2	rs41316003	0.007	A	G	-0.11	0.089	0.22	0.36	1.3×10^{-7}	28
	rs143103233	0.001	G	C	-0.065	0.23	0.77			
	rs150221602	0.001	C	G	-0.022	0.25	0.93			
JAK3	rs55778349	0.008	C	G	0.02	0.094	0.83			
TYK2	rs34536443	0.04	C	G	-0.39	0.039	1.5×10^{-23}			
	rs2304256[a]	0.28	A	C	-0.12	0.016	1.4×10^{-14}	0.25	6.4×10^{-168}	760
	rs12720356[b]	0.09	C	A	-0.15	0.025	2.4×10^{-9}	0.17	7.0×10^{-22}	
	rs35018800	0.008	A	G	-0.4	0.091	1.4×10^{-5}	0.33	6.3×10^{-4}	
	rs55762744	0.01	T	C	-0.2	0.076	0.007	-0.021	0.71	
	rs144960992	0.001	G	A	0.37	0.23	0.11			
	rs55886939	0.002	C	T	-0.23	0.19	0.21			
	rs55882956	0.002	A	G	-0.23	0.21	0.27			
	rs188833543	0.003	T	C	-0.15	0.14	0.29			
	rs139954108	0.0003	G	A	-1	1.5	0.49			

SNP = single-nucleotide polymorphism, MAF = minor allele frequency, EA = effect allele (aligned as the minor allele), NEA = non-effect allele, β_{T1D} = effect size for the risk of type 1 diabetes, SE_{T1D} = standard error for the effect size for the risk of type 1 diabetes, p_{T1D} = p-value for the risk of type 1 diabetes, β_{QTL} = beta coefficient for effect size for gene or protein expression, p_{QTL} = p-value for gene or protein expression. The values β_{QTL} for and p_{QTL} are provided only if the corresponding data were available. F-statistics are reported only for variants which were used in Mendelian randomization.

[a]rs2304256 is in very high LD with the lead eQTL rs34725611 (r^2 =1.00) and in moderate LD with the lead T1D risk variant rs144309607 (r^2=0.10).

[b]rs12720356 is in LD with the other TYK2 eQTL missense variant rs2304256 (r^2=0.29).

ESM Table 4. Linkage disequilibrium (LD) matrix (r^2) between four genetic variants near *TYK2* gene.

	**rs34536443**[a]	**rs12720356**[b]	**rs2304256**[c]	**rs34725611**[d]	**rs144309607**[e]
**rs34536443**[a]	1.00	<0.01	0.11	0.11	0.81
**rs12720356** [b]	<0.01	1.00	0.29	0.29	<0.01
**rs2304256**[c]	0.11	0.29	1.00	1.00	0.10
**rs34725611**[d]	0.11	0.29	1.00	1.00	0.11
**rs144309607**[e]	0.81	<0.01	0.10	0.11	1.00

[a]A known missense variant, which has previously reported to be associated with the risk of type 1 diabetes. [b]The second eQTL and type 1 diabetes risk variant among the missense variants in *TYK2* gene. [c]Top eQTL and type 1 diabetes risk variant among the missense variants in *TYK2* gene in this study. [d]The lead eQTL in the vicinity of *TYK2* gene. [e]The lead type 1 diabetes risk variant near *TYK2* gene. LD data based on the 1000 Genomes project European subsample.

ESM Table 5. Co-localisation results for single-cell expression levels and type 1 diabetes risk.

Gene	Cell type	H0	H1	H2	H3	H4	N	N_{SNP}
	Target level associated with genetic variants	No	Yes	No	Yes	Yes		
	Type 1 diabetes associated with genetic variants	No	No	Yes	Yes	Yes		
	Shared variant between target levels and type 1 diabetes	No	No	No	No	Yes		
IL2RA	Naïve/Immature B Cell	0	0	72.3	23.7	4	982	5,665
IL2RA	Memory B Cell	0	0	64	32.9	3.1	982	5,665
IL2RA	CD4 Effector memory/TEMRA	0	0	26.7	20.8	52.4	982	5,665
IL2RA	CD4 Naive/Central memory T cell	0	0	58.9	32.1	9	982	5,665
IL2RA	CD4 SOX4 T cell	0	0	60.6	36.2	3.3	982	5,665
IL2RA	CD8 Effector memory	0	0	0.2	0.4	99.4	982	5,665
IL2RA	CD8 Naive/Central memory T cell	0	0	25.7	20.9	53.4	982	5,665
IL2RA	CD8 S100B T cell	0	0	54.2	21.7	24.1	981	5,665
IL2RA	Classic Monocyte	0	0	69.9	26.1	4	969	5,665
IL2RA	Natural Killer Cell	0	0	63.3	33.9	2.8	928	5,665
IL2RA	Natural Killer Recruiting Cell	0	0	65.1	32.1	2.8	967	5,665
IL6R	Naïve/Immature B Cell	13	1.9	71.7	10.9	2.8	982	2,593
IL6R	Memory B Cell	4.6	10	25.8	58.2	1.1	982	2,593
IL6R	CD4 Effector memory/TEMRA	13	2.1	70.2	11.7	3.5	982	2,593
IL6R	CD4 Naive/Central memory T cell	12	2.3	65.2	13.2	7.7	982	2,593
IL6R	CD4 SOX4 T cell	12	2.3	68.8	12.8	3.8	982	2,593
IL6R	CD8 Effector memory	13	2	71.4	11.1	2.8	982	2,593
IL6R	CD8 Naive/Central memory T cell	11	3.1	63.7	17.5	4.4	982	2,593
IL6R	CD8 S100B T cell	12	2.6	66.1	14.4	5.2	981	2,593
IL6R	Dendritic Cell	12	3	65.2	17	3.2	982	2,593
IL6R	Classic Monocyte	12	2.3	65.6	13.1	7.2	969	2,593
IL6R	Non-classic Monocyte	13	1.8	72.6	10	2.7	932	2,593
IL6R	Natural Killer Cell	12	2.1	69.8	12	3.7	928	2,593
IL6R	Plasma Cell	12	2.2	67.9	12.5	5.3	643	2,593
IL6ST	Naïve/Immature B Cell	7.3	1.7	70.8	16.8	3.4	982	2,462
IL6ST	Memory B Cell	7.2	1.8	69.6	17.5	3.9	982	2,462
IL6ST	CD4 Effector memory/TEMRA	3.1	0.9	29.7	8.4	57.9	982	2,462
IL6ST	CD4 Naive/Central memory T cell	0	0.3	0	2.7	97	982	2,462
IL6ST	CD4 SOX4 T cell	5.7	2.9	55.1	28.1	8.1	982	2,462
IL6ST	CD8 Effector memory	6.7	1.1	64.5	11	16.8	982	2,462
IL6ST	CD8 Naive/Central memory T cell	0.4	0.4	3.5	3.3	92.4	982	2,462
IL6ST	CD8 S100B T cell	7.1	1.9	68.8	18.3	3.9	981	2,462
IL6ST	Dendritic Cell	5.7	3.3	55.5	31.5	4	982	2,462
IL6ST	Classic Monocyte	7	1.8	68.3	17.1	5.8	969	2,462
IL6ST	Non-classic Monocyte	7.7	1.3	75	12.3	3.7	932	2,462
IL6ST	Natural Killer Cell	7.6	1.3	73.9	12.7	4.4	928	2,462

ESM Table 5, continued

Gene	Cell type	H0	H1	H2	H3	H4	N	N_{SNP}
	Target level associated with genetic variants	No	Yes	No	Yes	Yes	No	Yes
	Type 1 diabetes associated with genetic variants	No	No	Yes	Yes	Yes	No	No
	Shared variant between target levels and type 1 diabetes	No	No	No	No	Yes	No	No
IL6ST	Natural Killer Recruiting Cell	7.9	1.1	76.3	10.8	3.8	967	2,462
IL6ST	Plasma Cell	6.3	1.3	61.1	12.1	19.2	643	2,462
TYK2	Naïve/Immature B Cell	0	0	77.1	18.5	4.4	982	3,710
TYK2	Memory B Cell	0	0	51	21.8	27.2	982	3,710
TYK2	CD4 Effector memory/TEMRA	0	0	77.9	17.8	4.4	982	3,710
TYK2	CD4 Naive/Central memory T cell	0	0	73.7	16.2	10.1	982	3,710
TYK2	CD4 SOX4 T cell	0	0	76.6	18.8	4.6	982	3,710
TYK2	CD8 Effector memory	0	0	69.4	24.9	5.8	982	3,710
TYK2	CD8 Naive/Central memory T cell	0	0	59.7	19	21.3	982	3,710
TYK2	CD8 S100B T cell	0	0	56.2	40.4	3.4	981	3,710
TYK2	Dendritic Cell	0	0	76.1	19.5	4.4	982	3,710
TYK2	Classic Monocyte	0	0	76.1	19.4	4.5	969	3,710
TYK2	Non-classic Monocyte	0	0	76.5	17.4	6.1	932	3,710
TYK2	Natural Killer Cell	0	0	77.9	16.7	5.4	928	3,710
TYK2	Natural Killer Recruiting Cell	0	0	71.8	23.5	4.7	967	3,710
TYK2	Plasma Cell	0	0	69.9	19.6	10.5	643	3,710

Data are reported as posterior probabilities in percentages. H0 to H4 refer to the different hypotheses in co-localisation (see Methods). N, the number of individuals in gene expression data; N_{SNP}, the number of variants in the co-localisation analysis.

ESM Table 6. Co-localisation results for bulk tissue expression levels and type 1 diabetes risk in disease-relevant tissues.

**Gene**	**Cell type**	**H0**	**H1**	**H2**	**H3**	**H4**	**N**	N_{SNP}
	Target level associated with genetic variants	No	Yes	No	Yes	Yes	No	Yes
	Type 1 diabetes associated with genetic variants	No	No	Yes	Yes	Yes	No	No
	Shared variant between target levels and type 1 diabetes	No	No	No	No	Yes	No	No
IL2RA	Spleen	0	0	25.7	69.5	4.7	211 to 227	9,756
IL6R	Pancreas	8.1	4.9	51.9	31.2	4	290 to 305	4,439
IL6R	Spleen	9.1	3.8	58.7	24.4	4	217 to 227	4,279
IL6ST	Pancreas	5.5	2.3	56.9	24.1	11.1	284 to 305	4,410
IL6ST	Spleen	5.9	2.5	60.7	26.1	4.8	211 to 227	4,318
TYK2	Pancreas	0	0	59.5	35.2	5.4	286 to 305	6,352
TYK2	Spleen	0	0	51.1	43.8	5	210 to 227	6,229

Data are reported as posterior probabilities in percentages. H0 to H4 refer to the different hypotheses in co-localisation (see Methods). N, the number of individuals in gene expression data; N_{SNP}, the number of variants in the co-localisation analysis.

References

1. Ferkingstad E, Sulem P, Atlason BA, et al. Large-scale integration of the plasma proteome with genetics and disease. Nat Genet 2021; 53: 1712–21.
2. Võsa U, Claringbould A, Westra H-J, et al. Large-scale cis- and trans-eQTL analyses identify thousands of genetic loci and polygenic scores that regulate blood gene expression. Nat Genet 2021; 53: 1300–10.
3. Chiou J, Geusz RJ, Okino ML, et al. Interpreting type 1 diabetes risk with genetics and single-cell epigenomics. Nature 2021; 594: 398–402.
4. Robertson CC, Inshaw JRJ, Onengut-Gumuscu S, et al. Fine-mapping, trans-ancestral and genomic analyses identify causal variants, cells, genes and drug targets for type 1 diabetes. Nat Genet 2021; 53(7):962–971.
